# Comparison of pulsed vacuum and ultrasound osmotic dehydration on drying of Chinese ginger (*Zingiber officinale* Roscoe): Drying characteristics, antioxidant capacity, and volatile profiles

**DOI:** 10.1002/fsn3.1103

**Published:** 2019-06-28

**Authors:** Kejing An, Daobang Tang, Jijun Wu, Manqin Fu, Jing Wen, Gengsheng Xiao, Yujuan Xu

**Affiliations:** ^1^ Sericulture and Agri‐Food Research Institute Guangdong Academy of Agricultural Sciences/Key Laboratory of Functional Foods Ministry of Agriculture/Guangdong Key Laboratory of Agricultural Products Processing Guangzhou China

**Keywords:** antioxidant activity, ginger (*Zingiber officinale* Roscoe), intermittent microwave & air‐drying, microstructure, PPO and POD activity, volatiles

## Abstract

The effects of pulsed vacuum osmotic dehydration (PVOD) and ultrasound osmotic dehydration (USOD) on drying characteristics and quality attributes of ginger were investigated. PVOD was subjected to pulsed vacuum at 13 kPa for 30 min, and USOD was subjected to ultrasound with the frequency of 40 kHz for 30 min. After PVOD and USOD treatments, the samples were then dried at intermittent microwave & air‐drying oven with an output of 700 W and temperature of 60°C to the final moisture content of 0.12 g water/g d.w. The results showed PVOD and USOD treatments could improve the total phenolic contents by −1.8% to 16.4%, total flavonoid content by 7.7%–18.7%, DPPH radical scavenging by 9.5%–12.2%, and ABTS+ antioxidant activity by 17.8%–27.4%, although they prolonged the later stages drying of ginger. Besides, the PVOD‐ and USOD‐pretreated dried samples had less brownings than the untreated‐dried samples which could be attributed to the inactivation of polyphenol oxidase (PPO) and peroxidase (POD). The PPO activity was significantly reduced in the PVOD and USOD ginger, whereas POD activity was decreased in USOD ginger but increased in PVOD ginger. Moreover, PVOD pretreatment also led to a better preservation of volatile profiles and cell structure than USOD treatment. Therefore, both PVOD and USOD are effective pretreatments for drying of ginger.

## 1 INTRODUCTION

The rhizome of the ginger plant (*Zingiber officinale*), which contains 85%–95% water, is susceptible to microbial spoilage and chemical deterioration (Mishra, Gauta, & Sharma, 2004). Drying is an effective technique that can be used to inhibit microbial growth and delay some deteriorative biochemical reactions in food materials (Shyam, 2006), which is often applied to ginger rhizome to extend its storage and transportation. Besides, it is also a fundamental processing to generate new products. Dried ginger can also be used as crude material for seasoning, flavoring tea, and ginger oleoresins.

Several predrying treatments, including osmotic dehydration (OD) (Mandala, Anagnostaras, & Oikonomou, 2005), ultrasound (Mothibe, Zhang, Nsor‐atindana, & Wang, 2011), high hydrostatic pressure (Yucel, Alpas, & Bayindirli, 2010), and carbonic maceration (Liu et al., 2014), have been reported to reduce the initial water content and to accelerate the drying process, resulting in improved quality of the final dried products with minimal cost. Different pretreatments exhibit diverse effects on final products. This can be achieved by modifying the structure as well as by inhibiting enzymatic activity and microorganisms, thus increases effective water diffusivity and quality attributes. OD at mild temperatures, which is considered minimal processing, preserves fresh‐like characteristics of fruits and vegetables, and can be used to obtain various products or ingredients for many food products. It was reported that OD could preserve attributes such as color, texture, and aroma, and reduce water activity, giving high‐moisture products extended shelf life (Dermesonlouoglou, Chalkia, & Taoukis, 2018; Moreno et al., 2011).

Pulsed vacuum osmotic dehydration (PVOD) consists of using OD at vacuum condition in the initial process followed by an OD at atmospheric pressure (Paes, Stringari, & Laurindo, 2007). By applying vacuum pressure, an outflow of internal gas or liquid from the tissue and the entrance of external solution are established via a hydrodynamic mechanism (HDM) that promotes water loss and the uptake of external solutes (Corrêa, Pereira, Vieira, & Hubinger, 2010). In ultrasound osmotic dehydration (USOD), ultrasonic waves could cause “sponge effect” and “cavitation effect,” which is considered to be responsible for the creation of microscopic channels in the fruits and vegetables, facilitating the removal of moisture (Awad, Moharram, Shaltout, Asker, & Youssef, 2012; Fuente‐Blanco, Sarabia, Acosta‐Aparicio, Blanco‐Blanco, & Gallego‐Juárez, 2006). Besides, deformation of porous solid materials caused by ultrasonic waves also reduces the diffusion boundary layer and increases the convective mass transfer in the fruit (Mothibe et al., 2011). Therefore, both PVOD and USOD have great potentials to increase the mass transfer of water loss and solutes gain during OD.

Pulsed vacuum osmotic dehydration (PVOD) and USOD have been reported to combine with air‐drying (Opalić et al., 2009), infrared drying (Brncic et al., 2010), and microwave drying (Yan et al., 2010). However, there were little literature reports about their combination with microwave & air‐drying. In microwave & air‐drying, microwave energy draws the inner moisture to the surface of the material and hot air removes the surface moisture. Microwave & air‐drying is thus highly efficient as the internal and external portions of the material are heated simultaneously (Zhang, Tang, Mujumdar, & Wang, 2006). In addition, if microwave energy is applied intermittently, the temperature and moisture levels of the target product are allowed to redistribute during off times, avoiding uneven heating and improving final product quality (Esturk, 2012).

However, the available reports mainly focus on the effects of PVOD and USOD on the mass transfer rate during osmotic dehydration and drying process (Deng & Zhao, 2008; Fernandes, Linhares, & Rodrigues, 2008; Fernandes & Rodrigues, 2008; Garcia‐Noguera et al., 2010; Kingsly, Goyal, Manikantan, & Ilyas, 2007; Xu, Zhang, Duan, Mujumdar, & Sun, 2009). To our knowledge, the studies on the effects of PVOD and USOD on quality attributes, for example, enzymes, antioxidant activity, and volatiles, are rather scarce.

Therefore, to further explore the different effects of PVOD and USOD on intermittent microwave & air‐drying and quality attributes of ginger, we carried out our study to: (a) investigate the effects of PVOD and USOD on the intermittent microwave & air‐drying of ginger; (b) determine the effects of PVOD and USOD on dried ginger quality attributes, including color, antioxidant activity, and volatiles; and (c) explore the mechanism for PVOD and USOD affecting mass transfer characteristics and quality attributes, such as oxidase activity and microstructure.

## 2 MATERIALS AND METHODS

### 2.1 Preparation of samples

Fresh, fully ripened ginger rhizomes (~13.28 g water/g d.w.) were purchased from a local market (Haidian District, Beijing, China). Voucher specimens were preserved at 4°C before experimentation. Raw ginger rhizomes were mechanically treated using a sharp cylindrical mold to produce circular slices, each with a thickness of 4.0 ± 0.2 mm and a diameter of 34.0 ± 2.0 mm. The initial moisture of ginger slices was determined with vacuum drying method (AOAC, 1995).

### 2.2 PVOD and USOD

Pulsed vacuum osmotic dehydration (PVOD) was carried out in a vacuum chamber connected to a vacuum pump (DZF‐5060; Shanghai Xinmiao Medical Apparatus and Instrument, Inc.). A beaker containing a ginger sample and the osmotic solution was placed inside the vacuum chamber and subjected to the following pulsed vacuum conditions: vacuum at 13 kPa for 15 min, release to normal pressure, and vacuum at 13 kPa for an additional 15 min. To test the USOD treatment, samples were placed in an ultrasonic chamber (KQ‐200DB; Kunshan Ultrasonic Apparatus Co.) for 30 min, with the operating frequency set to 40 kHz, output power set to 175 W, and intensity of 0.48 W/cm^2^. For both treatments, 30 g ginger slices were immersed into a ternary solution of 50% sucrose + 10% NaCl for 30 min, with a solution‐to‐material mass ratio of 4:1. Osmotic dehydration (OD) was conducted at 30 ± 1°C, based on previous results (An et al., 2012). Each assay was performed in triplicate.

### 2.3 Intermittent microwave & air‐drying following PVOD and USOD

Fresh, PVOD‐treated, and USOD‐treated ginger slices were dried at 60°C in a laboratory‐setup microwave & air‐drying oven, with an output of 700 W. Following the optimized intermittent microwave & air‐drying process (Tables S1 and S2), dehydrated samples were first dried at microwave pulse rate of 2 (5 s on:5 s off), until the moisture content was 1 g water/g d.w. The microwave pulse rate was then adjusted to 6 (5 s on:25 s off) to the end of drying (~0.12 g water/g d.w.). Under this condition, there was no hot pot and burning phenomena appeared. After drying, samples were ground into powder and stored at 4°C for further analysis.

### 2.4 Mathematical modeling of drying curves

Moisture ratio (MR) and drying rate (DR) can be expressed as following (Thakor, Sokhansanj, Sosulski, & Yannacopoulos, 1999):

(1) $$\text{MR}=\frac{M-M_{\text{e}}}{M_{0}-M_{\text{e}}}$$

(2) $$\text{DR}=-\frac{\text{d}M}{\text{d}t}=-\frac{M_{\text{d},i+1}-M_{\text{d},i}}{t_{i+1}-t_{i}}$$

where *M* is the instantaneous moisture content, dry basis; *M*_0_ is the initial moisture content, dry basis; *M*_e_ is the equilibrium moisture content, which can be assumed as zero when compared with *M*_0_. *M*_d__,i_ and *M*_d,i + 1_ are the moisture contents at times *t_i_* and *t_i_*_ + 1_, dry basis.

### 2.5 Color measurements

Colors of fresh, untreated‐dried, PVOD‐dried, and USOD‐dried ginger samples were measured using a Lab‐scan colorimeter (MS/S‐4500L; Hunter Associate Laboratory Inc.). The L* value is a measure of lightness, ranging from 0 (black) to +100 (white); a* value ranges from −100 (greenness) to + 100 (redness); and b* value ranges from −100 (blue) to +(100) (yellowness) (Gnanasekharan, Shewfelt, & Chinnan, 1992).

### 2.6 Measurement of polyphenol oxidase and peroxidase activity

Enzyme extraction. Enzyme extracts were made by 5 g of fresh, PVOD‐, and USOD‐treated ginger homogenized with 5 ml of 0.1 M acetate buffer (pH 5.5), which contained 4% (*w*/*v*) PVPP and 340% (*w*/*w*) PEG 6000.

Polyphenol oxidase (PPO) activity and peroxidase (POD) activity assay were performed according to Hernández and Cano (1998). The PPO activity and POD activity were determined by measuring the slope reaction, and the enzyme activity unit was defined as the change in absorbance per minute per gram of fresh weight of sample. Residual activity (RA) of enzyme was defined as:

(3) $$\text{RA}=\frac{A_{\text{t}}}{A_{0}}$$

where *A_t_* is the enzyme activity of ginger after pulsed vacuum or ultrasound treatment, and *A*_0_ is the initial enzyme activity of fresh ginger.

### 2.7 Preparation of ginger extracts

Extracts of fresh, untreated‐dried, PVOD‐dried, and USOD‐dried ginger were prepared according to the procedures described by Chan et al. (2008). Each sample was ultrasonically extracted three times, for 30 min each time, and then filtered. The extract was concentrated at 40°C in a rotary evaporator. After concentration, the extract was transferred to a volumetric flask and made up to the volume. The extract was stored at 4°C.

### 2.8 Determination of total phenolic content

The total phenolic contents of ginger extracts were determined with Folin–Ciocalteu assay according to Singleton, Orthofer, and Lamuela‐Raventos (1999). The amount of total phenolics was expressed as gallic acid equivalents (GAE, mg/g of dry sample).

### 2.9 Determination of total flavonoid content

The total flavonoid contents of ginger extracts were measured according to the method of Dewanto, Wu, Adom, and Liu (2002) with modifications. TFC was determined as rutin equivalents (mg/g of dry weight).

### 2.10 DPPH radical scavenging assay

DPPH radical scavenging assay on four types of ginger extracts was conducted according to Tohma et al. (2017) with modifications. Results were also expressed as ascorbic acid equivalent antioxidant capacity (AEAC).

Acid equivalent antioxidant capacity in mg ascorbic acid (AA)/g dry weight with following equation:

(4) $$\text{AEAC}\left(\text{mg}\;\text{ascorbic}\;\text{acid}/\text{g}\;\text{d}\text{.w.}\right)=\frac{{\text{IC}}_{50\left(\text{ascorbic}\;\text{acid}\right)}}{{\text{IC}}_{50\left(\text{sample}\right)}}\times 10^{5}$$

The IC_50 ascorbic acid_ used was determined to be 0.00387 mg/ml.

### 2.11 ABTS^+^ antioxidant activity

The ABTS antioxidant activity was carried out using the ABTS^+^ radical cation decolorization assay followed by the method of Sogi, Siddiq, Greiby, and Dolan (2013) with some modifications.

### 2.12 Determination of volatile flavor composition

The volatile components of fresh and dried ginger were isolated by solid‐phase microextraction (SPME) headspace GC‐MS method. The SPME manual device (Supelco Co.) was equipped with a fused silica fiber coated with polydimethylsiloxane (PDMS). 1.0 g of each dried ginger was placed in a 15‐ml vial and sealed. The fiber was inserted into the headspace for 30 min at 40°C; then, it desorbed at 250°C for 3 min in the injection port of an Agilent GC‐MS (7890A‐5975C).

Analysis of volatiles was according to the procedure described by Ding et al. (2012) with modifications. The aroma compounds were identified using an Agilent J&W DB‐5 column (30 m, 0.25 mm i.d., 0.25 μm film thickness). The oven temperature program was 3 min at 50°C, ramping to 120°C at 4°C/min, 8 min at 120°C, ramping to 200°C at 4ºC/min, 3 min at 200°C, ramping to 250°C at 10°C/min, and 3 min at 250°C. Helium was used as the carrier gas at a flow rate of 1.0 ml/min. The split ratio was 1:50. MS fragmentation was performed by electronic impact at 70 eV, with a source temperature of 230°C, a scanning rate of 1 scan s^−1^, and mass acquisition range of 35–550 Da. Compounds were preliminarily identified using the Wiley Online Library and the NIST library.

### 2.13 Microstructure of dried ginger

Microstructure changes of dried, PVOD‐dried, USOD‐dried ginger slices were analyzed using an optical microscope (DMI3000B; LEICA) and a scanning electron microscope (Phenom‐World BV). Optical microscope observation: The fresh and pretreated ginger slices were spread on the glass slide and observed directly. To obtain scanning electron micrographs, thin slices (~5 × 5 mm^2^) were glued on the metal stub and mounted onto the sample container. Each specimen was gold‐coated prior to *SEM* observation.

### 2.14 Statistical analysis

Experiment data and cluster analysis were conducted using Origin 8.0 (Microcal Software, Inc.) and SPSS 18.0. Significant differences among samples were identified using analyses of variance (ANOVAs) and Duncan's multiple‐range tests. We considered *p* < 0.05 statistically significant. All experiments were run in triplicate.

## 3 RESULTS AND DISCUSSION

### 3.1 The effects of PVOD and USOD pretreatment on drying of ginger

**Figure 1:**
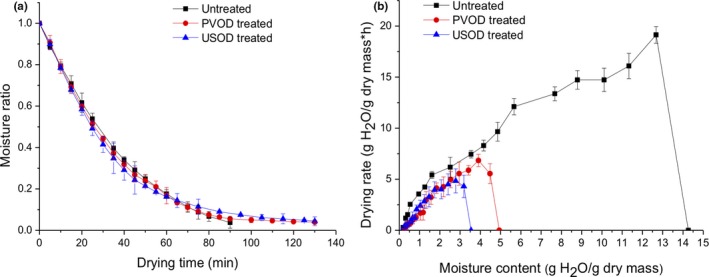
The (a) moisture ratio and (b) drying rate of untreated, pulsed vacuum osmotic dehydration (PVOD), and ultrasound osmotic dehydration (USOD) ginger samples during intermittent microwave & air‐drying. Points indicate the means of *X* independent experiments; error bars indicate standard deviations

The moisture ratios of the three types of dried samples (untreated, PVOD‐pretreated, and USOD‐pretreated) decreased exponentially with drying time (Figure 1a). USOD‐ and PVOD‐pretreated samples dried slightly faster than the untreated samples during the early stages of drying, but dried much more slowly than the untreated samples at the late stages of drying. This might be because more sugar was present in the USOD‐ and PVOD‐pretreated samples, and higher sugar concentrations increase the resistance to water diffusion (Mothibe et al., 2011). This was consistent with a previous study in USOD‐pretreated pineapple, whose decreased efficacy of diffusion during air‐drying was also thought to be due to the additional sugar incorporated in the fruit (Fernandes, Linhares et al., 2008). US‐pretreated pineapple, where the decreased efficacy of diffusion during air‐drying was also thought to be due to the additional sugar incorporated in the fruit (Fernandes, Linhares et al., 2008).

For the three types of dried samples, the drying rate either increased gradually or dropped suddenly; there were no periods of constant drying. This may have been because the ginger surfaces were constantly exposed to hot air, meaning that the internal diffusion rate was lower than the moisture evaporation rate on the surface. Therefore, it took on a falling‐rate period without constant rate period.

### 3.2 The effects of PVOD and USOD pretreatment on the colors of dried ginger

As Table 1 shows, the L* values of all dried samples were significantly lower than the L* value of fresh ginger, while a* values and b* values were significantly higher. This difference indicated that the dried samples had been subject to enzymatic and nonenzymatic brownings (Krokida, Maroulis, & Saravacos, 2001). However, the PVOD‐ and USOD‐dried samples had higher L* and b* values than the untreated‐dried samples and lower a* values. This indicated that the pretreated dried samples were less brown than the untreated‐dried samples. It is possible that vacuum impregnation reduces enzymatic browning due to the removal of oxygen from the intracellular spaces (An et al., 2013). UT may also effectively inhibit enzymatic browning. It has been shown that UT partially inactivated PPO and POD in apple cubes (Silva, Almeida, Rodrigues, & Fernandes, 2015). However, the effects of PVOD on PPO and POD activity in ginger remained unclear.

**Table 1:** Color of fresh ginger, untreated‐dried ginger, pulsed vacuum osmotic dehydration (PVOD)‐pretreated dried ginger, and ultrasound osmotic dehydration (USOD)‐pretreated dried ginger

	L*	a*	b*
Fresh	49.03 ± 0.92^c^	−1.99 ± 0.37^c^	17.62 ± 0.64^c^
Untreated dried	41.65 ± 5.34^d^	2.34 ± 0.25^a^	23.67 ± 2.65^b^
PVOD dried	64.61 ± 0.38^b^	1.52 ± 0.13^b^	30.13 ± 0.11^a^
USOD dried	74.31 ± 0.87^a^	1.20 ± 0.19^b^	31.57 ± 0.48^a^

Note

Values are means ± standard deviation (*n* = 3). In each column, different lowercase letters indicate significantly different values (*p* < 0.05; Duncan's test).

### 3.3 The effects of PVOD and USOD pretreatment on PPO and POD in ginger

**Figure 2:**
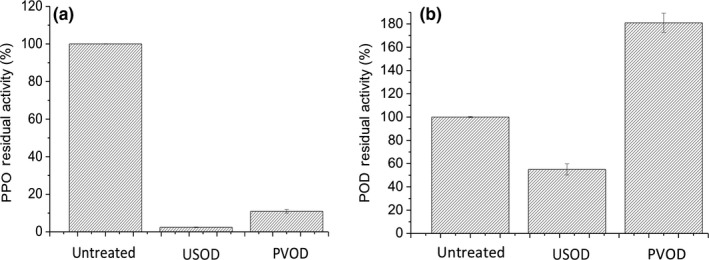
(a) Polyphenol oxidase (PPO) and (b) peroxidase (POD) activity in untreated, pulsed vacuum osmotic dehydration (PVOD)‐pretreated, and ultrasound osmotic dehydration (USOD)‐pretreated dried ginger. Bars show the means of X independent experiments; error bars indicate standard deviations

Polyphenol oxidase and POD are enzymes directly related to enzymatic browning in fruits and vegetables. PPO catalyzes the hydroxylation of monophenols and oxidation of *o*‐diphenols to *o*‐quinones; these molecules subsequently polymerize in the presence of oxygen to produce undesirable brown pigments (Barbagallo, Chisari, & Patané, 2012). POD activity results in food quality deteriorations, including flavor loss and biodegradation. POD is also involved in enzymatic browning, as diphenols may function as reducing substrates in fruits and vegetables (Jang & Moon, 2011). Therefore, the inactivation of PPO and POD allows a food product to retain more natural color and quality. PPO activity in the PVOD‐ and USOD‐dried ginger was significantly lower than in the untreated‐dried ginger (Figure 2a). POD activity in the USOD‐dried ginger was also significantly lower than in the untreated‐dried ginger, but POD activity was significantly higher in the PVOD‐dried ginger (Figure 2b). The cavitation produced by ultrasonic waves forms bubbles in the liquid that explosively collapse, leading to increase in localized pressure and temperature. This often leads to enzyme inactivation (Awad et al., 2012). Ultrasound had been reported to inactivate the tomato pectic enzyme, horseradish peroxidase, and orange PME (Soria & Villamiel, 2010). The hydrodynamic mechanism of PVOD is a consequence of the pressure gradients that result from the combined action of capillary flow and the pressure changes imposed on the porous structures of food tissue. The effect of pulsed vacuum was not so intense as that of ultrasound. Therefore, PVOD led to an activation of POD.

### 3.4 The effects of USOD and PVOD pretreatment on the total phenolic, flavonoid content, and antioxidant activity of dried ginger

Total flavonoid content, DPPH free radical scavenging, and ABTS antioxidant activity were significantly higher in all types of dried ginger, as compared to fresh ginger; total phenolic content was significantly higher in USOD‐treated ginger than in fresh ginger, but was significantly lower in untreated‐ and PVOD‐dried ginger (Table 2). Microwave radiation penetration may breakdown cellular constituents, making active components more accessible during extraction process (Toor & Savage, 2006), while the lower levels of phenolic compounds in the untreated‐ and PVOD‐dried samples might have been due to oxidative reactions during drying. As compared to fresh ginger, USOD treatment increased total flavonoid content by 18.68%, DPPH scavenging ability by 12.22%, and ABTS antioxidant activity by 27.35%. Similarly, PVOD treatment increased total flavonoids by 7.65%, DPPH scavenging ability by 9.47%, and ABTS antioxidant activity by 17.79%. These increases were likely due to the hydrodynamic mechanism effect of PVOD and the cavitation effect of USOD. The cavitations caused by ultrasonic waves may create microscopic channels and cause cellular disruption, reducing the diffusion boundary and increasing extraction efficiency (Soria & Villamiel, 2010). The inactivation of PPO and POD might also reduce the loss of active components. However, as compared to fresh ginger, PVOD treatment decreased total phenolic content by 1.78%, while USOD treatment increased total phenolic content by 16.36%. This may be because POD was activated after PVOD treatment, possibly resulting in some deterioration of phenols and other antioxidant components, though the hydrodynamic mechanism of PVOD prevented contact between the phenolic compounds and oxygen and inactivation of PPO reduced the oxidative reactions of the phenolic compounds.

**Table 2:** Total phenolic content (TPC), total flavonoid content (TFC), DPPH free radical scavenging, and ABTS antioxidant activity in fresh ginger, untreated‐dried ginger, pressure vacuum osmotic dehydration (PVOD)‐pretreated dried ginger, and ultrasound osmotic dehydration (USOD)‐pretreated dried ginger

	TPC^1^	TFC^2^	AEAC^3^	ABTS^4^
Fresh	11.97 ± 0.28^b^	13.49 ± 0.38^d^	100.49 ± 0.27^d^	64.56 ± 5.36^d^
Untreated dried	11.25 ± 0.67^c^	15.42 ± 0.30^c^	127.72 ± 2.01^c^	71.18 ± 1.52^c^
PVOD dried	11.05 ± 0.31^c^	16.60 ± 0.18^b^	139.81 ± 1.05^b^	83.84 ± 2.77^b^
USOD dried	13.09 ± 1.04^a^	18.30 ± 0.44^a^	143.33 ± 1.16^a^	90.65 ± 2.98^a^

Note

Values are means ± standard deviation (*n* = 3). In each column, different lowercase letters indicate significantly different values (*p* < 0.05; Duncan's test).

^1^ Expressed as mg GAE/g d.w.

^2^ Expressed as mg rutin/g d.w.

^3^ Expressed as mg ascorbic acid/g d.w.

^4^ Expressed as mg Trolox/g d.w.

### 3.5 The effects of USOD and PVOD pretreatment on the volatile components of dried ginger

There were 48 compounds extracted and identified in fresh ginger, including zingiberene (22.76%), β‐phellandrene (12.40%), β‐sesquiphellandrene (7.01%), geranial (14.50%), α‐curcumene (2.78%), and β‐bisabolene (3.25%) as the main compounds (Table S3). This was consistent with Huang, Wang, Chu, and Qin (2012) who also found zingiberene and β‐phellandrene were accounted for the odor of fresh ginger. In the untreated‐dried samples, 43 compounds were detected in IM&AD samples. The relative amounts of sesquiterpenes (zingiberene, β‐sesquiphellandrene, and curcumene) were considerably higher in untreated‐dried ginger as compared to the fresh sample, while the relative amounts of monoterpenes (β‐phellandrene, camphene) were significantly lower (Table S3). This result was consistent with our previous study (Ding et al., 2012) and may be due to the synthesis of short‐chain alkenes and the isomerization of similar compounds after long exposures to high temperatures and abundant oxygen. In PVOD‐ and USOD‐dried samples, we detected 40 and 51 volatile compounds, respectively (Table S3). It was shown that the use of osmotic pretreatments before strawberry freezing increased the formation of key compounds associated with fruit aromas, preventing loss of flavor (Torres, Talens, Carot, Chiralt, & Escriche, 2007). However, OD using more diluted solutions and shorter treatment times increased volatile production, while the use of highly concentrated osmotic solutions and longer treatment times caused volatile losses (Talens, Escriche, Martínez‐Navarrete, & Chiralt, 2002). Here, some of the key compounds in ginger aroma were increased after PVOD treatment, including zingiberene, β‐sesquiphellandrene, and curcumene, but some important aroma compounds were lost, including camphene, 3‐carene, and geranial. It may be that, although the PVOD hydrodynamic mechanism prevented contact between oxygen and substrates, the higher POD activity also caused some flavor deterioration. In the USOD‐dried samples, many new components were generated, including 1, 3, 8‐p‐menthatriene; 2, 6‐dimethyl‐1, 3, 5, 7‐octatetraene, E, E‐; naphthalene, 1, 2, 3, 5, 6, 8a‐hexahydro‐4, 7‐dimethyl‐1‐(1‐methylethyl)‐,(1S‐cis)‐; and bornyl acetate (Table S3). Compared to the PVOD‐dried samples, the USOD‐dried samples had fewer alkanes and more alkenes and alcohols. This may be because the acoustical cavitation of UT increased the decomposition, isomerization, and oxidation of volatile components.

**Figure 3:**
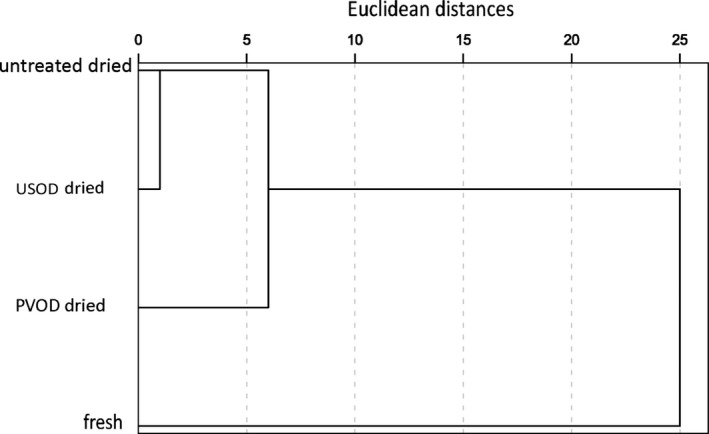
Ward connection spectrum dendrogram showing the similarity of volatile components in fresh, untreated‐dried, pulsed vacuum osmotic dehydration (PVOD)‐pretreated dried, and ultrasound osmotic dehydration (USOD)‐pretreated dried ginger samples

Cluster analysis was carried out to determine the correlations among the different drying methods with respect to volatile compounds. Samples separated by greater Euclidean distances had more dissimilar volatile compounds. The untreated‐dried samples had the shortest Euclidean distance to the fresh samples, followed by the PVOD‐ and USOD‐dried samples (Figure 3; Table S4). USOD‐dried samples were the most dissimilar to fresh samples, while PVOD‐dried samples were more similar to the untreated‐dried samples (Figure 3; Table S4).

### 3.6 The effects of USOD and PVOD pretreatment on microstructure of dried ginger

**Figure 4:**
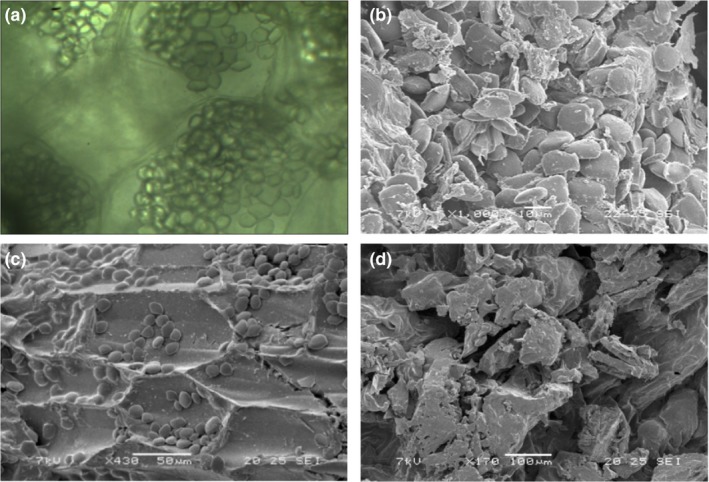
Surface morphology of ginger. (a) Fresh (light microscope: ×630). (b) Untreated dried (scanning electron microscope [*SEM*]: ×1,000). (c) Pulsed vacuum osmotic dehydration (PVOD)‐pretreated dried (*SEM*: ×430). (d) Ultrasound osmotic dehydration (USOD)‐pretreated dried (*SEM*: ×470)

The structure of fresh ginger was clear when examined under light microscopy (Figure 4a): Cells were evenly distributed, the pectin‐laced walls were intact, and abundant starch grains were present. After drying without pretreatment, parenchyma cells were severely damaged, and the starch grains were scattered all over the tissue after drying (Figure 4b). After PVOD treatment, the basic structures of the ginger cell were retained and starch grains were preserved intact; however, small, irregular intercellular spaces were present (Figure 4c). After USOD treatment, parenchyma cell structure was severely damaged, and the starch grains were not well preserved. This may have been due to the formation of microscopic channels in the ginger, which resulted in the loss of cellular adhesion, and lead to the production of large intercellular spaces. This was also proved by other researchers that USOD treatment caused substantial turgor loss and noticeable tissue damage (Fernandes & Rodrigues, 2008).

## 4 CONCLUSIONS

Pulsed vacuum osmotic dehydration (PVOD) and USOD pretreatment profoundly affected the drying kinetics and quality attributes of ginger. PVOD and USOD treatment prolonged drying time because increased solute penetration blocked internal porosity. Both PVOD and USOD significantly reduced PPO activity, inhibiting enzymatic browning and increasing total flavonoid content, DPPH radical scavenging, and ABTS+ antioxidant activity in the dried ginger. USOD also significantly reduced POD activity, increasing the total phenolic content of the dried ginger. PVOD treatment conserved volatile profiles and preserved cell structure more effectively than did USOD treatment. Despite these differences, it was clear that the pretreated dried ginger was higher quality than the untreated‐dried ginger. Therefore, PVOD and USOD are both effective pretreatments for drying of ginger.

## CONFLICT OF INTEREST

The authors do not have any conflicting interests.

## ETHICAL APPROVAL

This study does not involve any human or animal testing.

## INFORMED CONSENT

None.
