# Supplementary material for: Comparison of pulsed vacuum and ultrasound osmotic dehydration on drying of Chinese ginger (Zingiber officinale Roscoe): Drying characteristics, antioxidant capacity, and volatile profiles
Source: Food Sci Nutr. 2019 Jun 28;7(8):2537–45. doi: 10.1002/fsn3.1103 (PMC6694637; doi:10.1002/fsn3.1103)
Supplement: Supplementary file 1 [file FSN3-7-2537-s001.docx]

Table S1 Factors and levels in intermittent microwave-air drying orthogonal experiment

| Factor | Levels | | | |
| --- | --- | --- | --- | --- |
|  | -1 | 0 | 1 |  |
| Microwave pulsed ratio of stage 1 | 2 (5 s/5s, t_on_/t_off_) | 3 (5 s/10 s, t_on_/t_off_ ) | 3.4(5 s/12 s, t_on_/t_off_) | |
| Water content of conversion point | 1.0 | 2.5 | 4.0 | |
| Microwave pulsed ratio of stage 2 | 4 (5 s/15 s, t_on_/t_off_) | 5 (5 s/20 s, t_on_/t_off_) | 6 (5s/25, t_on_/t_off_) | |

Table S2 Orthogonal test of optimization of hot air with intermittent microwave drying process

| Index | Run | A | B | C | DT | DE | ΔE* | TPC |
| --- | --- | --- | --- | --- | --- | --- | --- | --- |
| 1 | 1(2) | 1(1.0) | 1(4) | 91 | 3.57 | 14.472 | 15.06 |  |
|  | 2 | 1(2) | 2(2.5) | 2(5) | 127 | 5.25 | 18.863 | 13.97 |
|  | 3 | 1(2) | 3(4.0) | 3(6) | 153 | 6.02 | 18.494 | 12.93 |
|  | 4 | 2(3) | 1(1.0) | 2(5) | 142 | 5.70 | 16.208 | 14.37 |
|  | 5 | 2(3) | 2(2.5) | 3(6) | 121 | 4.64 | 16.184 | 12.54 |
|  | 6 | 2(3) | 3(4.0) | 1(4) | 149 | 5.80 | 20.396 | 11.48 |
|  | 7 | 3(3.4) | 1(1.0) | 3(6) | 143 | 5.47 | 17.978 | 13.47 |
|  | 8 | 3(3.4) | 2(2.5) | 1(4) | 188 | 6.96 | 21.700 | 10.35 |
|  | 9 | 3(3.4) | 3(4.0) | 2(5) | 151 | 6.11 | 19.406 | 12.48 |
| DT(min) | K1 | 371 | 376 | 428 | A＞B＞C A_1_B_1_C_3_ | | | |
|  | K2 | 412 | 436 | 420 |  |  |  |  |
|  | K3 | 482 | 453 | 417 |  |  |  |  |
|  | R | 111 | 77 | 11 |  |  |  |  |
| DE(kJ/kg) H_2_O) | K1 | 14.84 | 14.74 | 16.33 | A＞B＞C A_1_B_1_C_3_ | | | |
|  | K2 | 16.14 | 16.85 | 17.06 |  |  |  |  |
|  | K3 | 18.54 | 17.93 | 16.13 |  |  |  |  |
|  | R | 3.7 | 3.19 | 0.93 |  |  |  |  |
| ΔE* | K1 | 51.83 | 48.66 | 56.57 | B＞A＞C A_1_B_1_C_3_ | | | |
|  | K2 | 52.79 | 56.75 | 54.48 |  |  |  |  |
|  | K3 | 59.08 | 58.30 | 52.66 |  |  |  |  |
|  | R | 7.25 | 9.64 | 3.91 |  |  |  |  |
| TPC(mg/g) | K1 | 41.96 | 42.9 | 36.89 | B＞A＞C A_1_B_1_C_2_ | | | |
|  | K2 | 38.39 | 36.86 | 40.82 |  |  |  |  |
|  | K3 | 36.3 | 36.89 | 38.94 |  |  |  |  |
|  | R | 5.66 | 6.04 | 3.93 |  |  |  |  |

Note: DT, drying time, min; DE, drying efficiency, kJ/kg H_2_O; ΔE*, colour difference；TPC，total phenolic content, mg/g

Table S3 Comparison of volatile components of intermittent microwave assisted air dried gingers after different osmotic pretreatments

| Peak no. | Retention time (min) | Compounds | Molecular  formular | Fraction (%) | | | |
| --- | --- | --- | --- | --- | --- | --- | --- |
|  |  |  |  | Fresh | Untreated dried | PVOD  dried | UTOD dried |
| Alkenes | | | | | | | |
| 1 | 12.716 | 1R-α-Pinene | C_10_H_16_ | 1.55±0.01 | ----- | 0.07±0.015 | ----- |
| 2 | 13.151 | 1s-α-Pinene | C_10_H_16_ | ------ | 0.16±0.01 | ----- | 0.11±0.002 |
| 3 | 13.733 | camphene | C_10_H_16_ | 5.13±0.02 | 0.92±0.02 | 0.34±0.02 | 0.56±0.003 |
| 4 | 14.81 | β-Pinene | C_10_H_16_ | 0.16±0.01 | 0.04±0.003 | 0.02±0.002 | 0.03±0.01 |
| 5 | 15.239 | β-Myrcene | C_10_H_16_ | 0.98±0.02 | 0.21±0.03 | 0.1±0.012 | 0.15±0.02 |
| 6 | 15.822 | α-Phellandrene | C_10_H_16_ | 0.8±0.15 | 0.11±0.001 | 0.08±0.002 | 0.09±0.001 |
| 7 | 16.275 | 1,3-Cyclohexadiene, 1-methyl-4-(1-methylethyl)- | C_10_H_16_ | 0.08±0.01 | ----- | ----- | ----- |
| 8 | 16.616 | 1,3,8-p-Menthatriene | C_10_H_14_ | ----- | ----- | 0.01±0.001 | 0.9±0.003 |
| 9 | 16.774 | Bicyclo[3.1.0]hexane,4-methylene-1-(1-methylethyl)- | C_10_H_16_ | ----- | 1.39±0.06 | 0.57±0.04 | ----- |
| 10 | 16.786 | β-Phellandrene | C_10_H_16_ | 12.4±0.25 | ----- | ----- | ----- |
| 11 | 17.88 | 1,4-Cyclohexadiene, 1-methyl -4-(1-methylethyl)- | C_10_H_16_ | 0.09±0.01 | ----- | ----- | ----- |
| 12 | 18.998 | Cyclohexene,1-methyl-4-(1-methylethylidene)- | C_10_H_16_ | 0.39±0.02 | 0.14±0.05 | ----- | 0.11±0.032 |
| 13 | 19.004 | (+)-4-Carene | C_10_H_16_ | ----- | ----- | 0.08±0.002 | ----- |
| 14 | 19.339 | 1,3,6-Octatriene,3,7-dimethyl-, (Z) | C_10_H_16_ | 0.14±0.01 | ------ | ----- | 0.35±0.002 |
| 15 | 19.345 | 3-Carene | C_10_H_16_ | ------ | 0.35±0.14 | ----- | ----- |
| 16 | 20.298 | 3-Carene | C_10_H_16_ | ------ | 0.03±0.002 | 0.03±0.0023 | 0.03±0.001 |
| 17 | 26.18 | 2,6-Dimethyl-1,3,5,7-octatetraene,E,E- | C_10_H_14_ | ----- | ----- | ----- | 0.3±0.002 |
| 18 | 31.398 | 1,3-Cyclohexadiene, 1-methyl-4-(1-methylethyl)- | C_10_H_16_ | 0.08±0.01 | ----- | ----- | ----- |
| 19 | 31.403 | Bicyclo[4.1.0]hept-2-ene,3,7,7-trimethyl- | C_10_H_16_ | ----- | ----- | 0.18±0.001 | ----- |
| 20 | 31.415 | Cyclohexene,4-ethenyl-4-methyl-3-(1-methylethenyl)-1-(1-methylethyl)-, (3R-trans)- | C_15_H_24_ | ----- | 0.15±0.02 | ----- | 0.16±0.004 |
| 21 | 32.091 | 2,6-Octadiene,2,6-dimethyl- | C_10_H_18_ | 0.38±0.02 | 0.25±0.001 | 0.23±0.004 | 0.27±0.03 |
| 22 | 33.05 | (+)-Cycloisosativene | C_15_H_24_ | 0.08±0.01 | 0.53±0.01 | 0.45±0.02 | ----- |
| 23 | 33.05 | 1,2,4-Metheno-1H-indene,octahydro-1,7a-dimethyl-5-(1-methylethyl)-,[1S-(1a,2a,3aβ,4a,5a,7aβ.,8S*)] | C_15_H_24_ | ----- | ------ | ----- | 0.54±0.06 |
| 24 | 33.538 | Copaene | C_15_H_24_ | ------ | 1.02±0.02 | ----- | 1.00±0.005 |
| 25 | 33.538 | Alpha-Cubebene | C_15_H_24_ | 0.12±0.01 | ------ | 0.89±0.03 | ----- |
| 26 | 33.703 | 3-Carene | C_10_H_16_ | 4.05±0.04 | 0.52±0.02 | 0.47±0.01 | 0.52±0.02 |
| 27 | 34.3 | Cyclohexane,1-ethenyl-1-methyl-2, 4-bis(1-methylethenyl)-, [1S-(1.al  pha.,2.beta.,4.beta.)]- | C_15_H_24_ | 0.28±0.02 | 0.91±0.01 | 0.97±0.06 | 0.98±0.01 |
| 28 | 34.855 | Bicyclo[3.1.1]hept-2-ene,2,6-dimethyl-6-(4-methyl-3-pentenyl)- | C_15_H_24_ | 0.11±0.002 | ------ | ----- | ----- |
| 29 | 34.879 | 1,3-Cyclohexadiene,5-(1,5-dimethyl-4-hexenyl)-2-methyl-,[S-(R*,S*)]- | C_15_H_24_ | ------ | 0.48±0.06 | 0.44±0.04 | 0.47±0.02 |
| 30 | 35.679 | Caryophyllene | C_15_H_24_ | ----- | 0.14±0.02 | 0.15±0.01 | 0.15±0.01 |
| 31 | 36.103 | 1H-Cyclopenta[1,3]cyclopropa[1,2]benzene,  octahydro-7-methyl-3-methylene-4-(1-methylethyl)-,[3aS-(3a.alpha.,3b.beta.,4.beta.,7.alpha.,7aS*)] | C_15_H_24_ | ----- | 0.07±0.01 | ----- | ----- |
| 32 | 36.22 | gamma.-Elemene | C_16_H_26_ | ------ | ----- | 0.45±0.02 | 0.2±0.015 |
| 33 | 36.221 | 1,5-Cyclodecadiene,1,5-dimethyl-8-(1-methylethylidene)-,(E,E) | C_15_H_24_ | 0.14±0.01 | ----- | ----- | ----- |
| 34 | 36.744 | 1H-3a,7-Methanoazulene, 2,3,4,7,8,8a-hexahydro-3,6,8,8-tetramethyl-,[3R-(3.alpha.,3a.beta.,7.beta.,8a.alpha.)]- | C_15_H_24_ | ----- | ----- | ----- | 0.05±0.001 |
| 35 | 36.838 | Naphthalene, 1,2,3,5,6,8a-hexahydr o-4,7-dimethyl-1-(1-methylethyl)-,(1S-cis)- | C_15_H_24_ | ----- | ----- | ----- | 0.03±0.005 |
| 36 | 36.938 | 1H-Cycloprop[e]azulene,1a,2,3,4,4a,5,6,7b-octahydro-1,1,4,7-tetramethyl-,[1aR-(1a.alpha.,4.alpha.,4a.beta.,7b.alpha.)]- | C_15_H_24_ | ----- | 0.09±0.003 | 0.11±0.004 | 0.09±0.001 |
| 37 | 37.02 | Copaene | C_15_H_24_ | ----- | ----- | ----- | 0.04±0.01 |
| 38 | 37.12 | 1,6,10-Dodecatriene,7,11-dimethyl-3-methylene-, (Z)- | C_15_H_24_ | 0.15±0.02 | 0.73±0.02 | 0.71±0.01 | 0.71±0.01 |
| 39 | 37.502 | 1H-Cycloprop[e]azulene,decahydro-1,1,7-trimethyl-4-methylene-, [1aR-(1a.alpha.,4a.beta.,7.alpha.,7a.beta.,7b.alpha.)]- | C_15_H_24_ | ----- | ----- | ----- | 0.53±0.01 |
| 40 | 37.503 | 1H-Cycloprop[e]azulene,decahydro-1,1,7-trimethyl-4-methylene-,[1aR-(1a.alpha.,4a.beta.,7.alpha.,7a.beta.,7b.alpha.)]- | C_15_H_24_ | 0.13±0.01 | 0.53±0.07 | 0.48±0.06 | 0.22±0.004 |
| 41 | 38.302 | Curcumene | C_15_H_22_ | 2.78±0.02 | 5.82±0.16 | 5.98±0.20 | 5.2±0.33 |
| 42 | 38.467 | Naphthalene, 1,2,3,5,6,8a-hexahydro-4,7-dimethyl-1-(1-methylethyl)-,(1S-cis)- | C_15_H_24_ | ----- | ----- | ----- | 0.33±0.03 |
| 43 | 38.473 | Isoledene | C_15_H_24_ | 0.09±0.003 | 0.35±0.05 | ----- | ----- |
| 44 | 38.555 | Naphthalene,decahydro-4a-methyl-1-methylene-7-(1-methylethenyl)-,[4aR-(4a.alpha.,7.alpha.,8a.beta.)] | C_15_H_24_ | ----- | ------ | ----- | 0.17±0.01 |
| 45 | 38.891 | Zingiberene | C_15_H_24_ | 22.76±0.45 | 42.2±1.54 | 41.06±2.40 | 38.82±0.88 |
| 46 | 39.026 | Naphthalene,1,2,3,4,4a,5,6,8a-octahydro-7-methyl-4-methylene  -1-(1-methylethyl)-,(1.alpha.,4a.beta.,8a.4.42alpha.)- | C_15_H_24_ | ----- | 2.82±0.12 | 2.73±0.14 | 2.93±0.20 |
| 47 | 39.244 | a-Farnesene | C_15_H_24_ | 2.58±0.06 | 3.91±0.11 | ----- | 4.66±0.14 |
| 48 | 39.332 | β-bisabolene | C_15_H_24_ | 3.25±0.32 | 5.93±1.22 | 10.77±1.60 | 5.51±0.60 |
| 49 | 39.556 | Bicyclo[4.4.0]dec-1-ene, 2-isopropyl-5-methyl-9-methylene- | C_15_H_24_ | 0.17±0.01 | ----- | ----- | ----- |
| 50 | 39.567 | (+)-Epi-bicyclosesquiphellandrene | C_15_H_24_ | ----- | 0.42±0.01 | 0.48±0.02 | 0.48±0.05 |
| 51 | 39.938 | β-sesquiphellandrene | C_15_H_24_ | 7.01±0.03 | 11.24±0.78 | 11.35±1.35 | 10.71±1.01 |
| 52 | 40.473 | Naphthalene,1,2,4a,5,6,8a-hexahydro-4,7-dimethyl-1-(1-methylethyl)-,[1R-(1.alpha.,4a.alpha.,8a.alpha.)] | C_15_H_24_ | ------ | 0.04±0.002 | 0.13±0.001 | 0.08±0.015 |
| 53 | 40.873 | 1H-Cycloprop[e]azulene, decahydro-1,1,7-trimethyl-4-methylene-,[1aR-(1a.alpha.,4a.beta.,7.alpha.,7a.beta.,7b.alpha.)]- | C_15_H_24_ | ----- | ----- | ----- | 0.04±0.003 |
| 54 | 41.243 | gamma.-Elemene | C_16_H_26_ | 0.2±0.02 | ----- | ----- | 0.19±0.01 |
| Aromatic hydrocarbon | | | | | | | |
| 1 | 16.593 | Benzene, 1-methyl-2-(1-methylethyl)- | C_10_H_14_ | 0.09±0.01 | ----- | ----- | ----- |
| 2 | 16.61 | Benzene,  1-methyl-3-(1-methylethyl)- | C_10_H_14_ | ----- | ------ | ----- | 0.02±0.004 |
| 3 | 31.091 | Dodecane, 2,7,10-trimethyl- | C_15_H_32_ | ----- | ----- | 0.04±0.001 | ----- |
| Alkanes | | | | | | | |
| 1 | 11.639 | Tricyclo[2.2.1.0(2,6)]heptane, 1,7,7-trimethyl | C_10_H_16_ | 0.08±0.01 | ----- | ----- | ----- |
| 2 | 14.634 | Cyclohexene, 4-methylene-1-(1-methylethyl)- | C_10_H_16_ | 0.12±0.02 | ----- | ----- | ----- |
| 3 | 19.962 | Bicyclo[2.2.1]heptane,2-methoxy-1,7,7-trimethyl- | C_11_H_20_O | ------ | 0.06±0.003 | ----- | ----- |
| 4 | 38.561 | Naphthalene,1,2,3,4,4a,5,6,8a-octahydro-4a,8-dimethyl-2-(1-methylethenyl)-,[2R-(2.alpha.,4a.alpha.,8a.beta.)] | C_15_H_24_ | ----- | 0.14±0.001 | ----- | ----- |
| 5 | 39.032 | 1Naphthalene,1,2,4a,5,6,8a-hexahydro-4,7-dimethyl-1-(1-methylethyl)- | C_15_H_24_ | 0.9±0.02 | ------ | ----- | ----- |
| 6 | 41.244 | Naphthalene,1,2,3,4,4a,5,6,8a-octahydro-4a,8-dimethyl-2-(1-methylethenyl)-,[2R-(2.alpha.,4a.alpha.,8a.beta.) | C_15_H_24_ | ----- | ----- | 0.04±0.002 | ----- |
| 7 | 41.244 | Naphthalene,1,2,3,4,4a,5,6,8a-octahydro-4a,8-dimethyl-2-(1-methylethenyl)-,[2R-(2.alpha.,4a.alpha.,8a.beta.) | C_15_H_24_ | ----- | ----- | 0.23±0.015 | ----- |
| 8 | 41.249 | Cyclohexane,1-ethenyl-1-methyl-2-(1-methylethenyl)-4-(1-methylethylidene)- | C_15_H_24_ | ----- | 0.11±0.001 | ----- | ----- |
| Alcohol | | | | | | | |
| 1 | 10.697 | 2-Heptanol | C_7_H_16_O | 0.15±0.04 | ----- | ----- | ----- |
| 2 | 16.863 | Eucalyptol | C_10_H_18_O | 5.33±0.12 | 2.51±0.01 | 1.27±0.003 | 2.08±0.013 |
| 3 | 21.192 | Bicyclo[2.2.1]heptan-2-one, 1,7,7- trimethyl- (1R)- | C_10_H_16_O | ----- | 0.09±0.02 | ----- | ----- |
| 4 | 21.509 | Isoborneol | C_10_H_18_O | 0.06±0.01 | ------ | ----- | ----- |
| 5 | 22.004 | Borneol | C_10_H_18_O | 2.15±0.05 | 0.99±0.03 | 0.73±0.02 | 0.82±0.01 |
| 6 | 22.256 | Tricyclo[4.3.1.1(3,8)]undecan-1-ol Methanone, cyclobutyl-1H-imidazol- 4-yl- | C_11_H_18_O | ----- | ----- | ----- | 0.03±0.002 |
| 7 | 22.48 | 3-Cyclohexen-1-ol,4-methyl-1-(1-methylethyl) | C_10_H_18_O | 0.31±0.11 | ------ | ----- | ----- |
| 8 | 22.527 | Cyclohexanol,5-methyl-2-(1-methylethenyl)-,(1.alpha.,2.beta.,5.alpha.) | C_10_H_18_O | ----- | ------ | ----- | 0.23±0.45 |
| 9 | 23.075 | 3-Cyclohexene-1-methanol,.α,α-4-trimethyl- | C_10_H_18_O | 1.03±0.01 | 0.55±0.004 | 0.63±0.003 | 0.71±0.04 |
| 10 | 26.048 | 2,6-Octadien-1-ol,3,7-dimethyl-(Z)- | C_18_H_24_O_2_ | 2.11±0.20 | ------ | ----- | ----- |
| Aldehydes | | | | | | | |
| 1 | 15.256 | Octanal | C_8_H_16_O | 0.11±0.01 | ----- | ----- | ----- |
| 2 | 21.333 | 7-Octenal, 3,7-dimethyl- | C_10_H_18_O | ------ | 0.16±0.03 | ----- | ----- |
| 3 | 23.356 | Bicyclo[3.1.1]hept-2-ene-2-carboxaldehyde, 6,6-dimethyl- | C_10_H_14_O | 0.11±0.02 | ----- | ----- | 0.08±0.002 |
| 4 | 23.362 | (1R)-(-)-Myrtenal | C_10_H_14_O | ----- | 0.05±0.007 | ----- | ----- |
| 5 | 24.633 | 6-Octen-1-ol, 3,7-dimethyl-(R)- | C_10_H_20_O | 0.23±0.04 | ------ | 0.09±0.005 | 0.1±0.01 |
| 6 | 25.409 | Geranial-(Z) | C_10_H_16_O | 2.82±0.12 | 3.14±0.24 | 4.31±0.47 | 3.91±0.36 |
| 7 | 27.103 | Geranial | C_10_H_16_O | 14.5±1.02 | 7.31±0.78 | 9.38±1.45 | 10.22±0.06 |
| Ketone | | | | | | | |
| 1 | 21.186 | Camphor | C_10_H_16_O | 0.15±0.01 | ----- | 0.05±0.01 | 0.07±0.005 |
| 2 | 28.515 | 2-Undecanone | C_11_H_22_O | 0.35±0.01 | 0.08±0.02 | 0.1±0.01 | 0.17±0.03 |
| Esters | | | | | | | |
| 1 | 28.174 | Bicyclo[2.2.1]heptan-2-ol,1,7,7-trimethyl-,acetate,(1S-endo)- | C_12_H_20_O_2_ | 0.42±0.02 | 0.52±0.04 | 0.34±0.014 | ----- |
| 2 | 28.185 | Bornyl acetate | C_12_H_20_O_2_ | ----- | ------ | ----- | 0.49±0.07 |

Values are means ± standard deviation (n = 3).

Table S4 Approximation matrix of volatile components among different ginger samples: fresh, untreated dried, pressure vacuum osmotic dehydrated (PVOD)-pretreated dried, and ultrasound osmotic dehydrated (UTOD)-pretreated dried.

| dehydration  methods | The average Euclidean distance | | | | |
| --- | --- | --- | --- | --- | --- |
|  | Fresh | AD&IM | PVOD+AD&IM | | UTOD+AD&IM |
| Fresh | 0.00 | 419.36 | 452.02 | 662.76 | |
| Untreated dried | 419.36 | 0.00 | 26.13 | 46.88 | |
| PVOD dried | 452.02 | 26.13 | 0.00 | 53.43 | |
| UTOD dried | 662.76 | 46.88 | 53.43 | 0.00 | |
